# Association between leukocyte telomere length and COVID-19 severity

**DOI:** 10.1186/s43042-023-00415-z

**Published:** 2023-05-29

**Authors:** Ata Mahmoodpoor, Sarvin Sanaie, Maqsoud Eskandari, Nazila Behrouzi, Majid Taghizadeh, Faranak Roudbari, Babak Emamalizadeh, Nasim Sohrabifar, Somayeh Kazeminasab

**Affiliations:** 1 Department of Anesthesiology and Intensive Care, Faculty of Medicine, Tabriz University of Medical Sciences, Tabriz, Iran; 2 Research Center of Psychiatry and Behavioral Sciences, Aging Research Institute, Tabriz University of Medical Sciences, Tabriz, Iran; 3 Faculty of Natural Sciences, Tabriz University, Tabriz, Iran; 4 Department of Medical Genetics, Faculty of Medicine, Tabriz University of Medical Sciences, Tabriz, Iran; 5 Cardiovascular Research Center, Shahid Beheshti University of Medical Sciences, Tehran, Iran

**Keywords:** Telomere, Leukocyte telomere length, SARS-CoV-2, COVID-19, Severity

## Abstract

**Background:**

Inter-individual variations in the clinical manifestations of SARS-CoV-2 infection are among the challenging features of COVID-19. The known role of telomeres in cell proliferation and immune competency highlights their possible function in infectious diseases. Variability in telomere length is an invaluable parameter in the heterogeneity of the clinical presentation of diseases.

**Result:**

In this study, our aim was to investigate the possible association between leukocyte telomere length (LTL) and COVID-19 severity. LTL was measured in 100 patients with moderate and severe forms of COVID-19 using the quantitative PCR (q-PCR) method. Statistical analysis confirmed a strong inverse correlation between relative LTL and COVID-19 severity.

**Conclusions:**

These findings suggest that LTL can be a useful parameter for predicting disease severity in patients, as individuals with short telomeres may have a higher risk of developing severe COVID-19.

**Supplementary Information:**

The online version contains supplementary material available at 10.1186/s43042-023-00415-z.

## Introduction

Severe acute respiratory syndrome coronavirus (SARS-CoV)-2 first emerged in Wuhan City, China, in late December 2019. The World Health Organization announced “COVID-19” as the name of this dangerous and deadly disease. Within a short period of time, the COVID-19 pandemic has spread with alarming speed and threatens millions of people around the world [1]. SARS-CoV-2 is an enveloped virus with a positive-sense, single-stranded RNA genome of 29.8 kb. There are different scenarios for the origin of the virus; however, phylogenetic analyses have shown that this virus has likely originated by sequential recombination of bat SARS-CoVs [2, 3]. Epidemiological investigations have demonstrated that SARS-CoV-2 affects all ages, but elderly people with underlying diseases are more susceptible. A systematic review of Chinese cases reported a 3.2% case fatality rate, ranging from 2 to 4% for COVID-19. However, higher age and preexisting comorbid conditions were described as the main risk factors for mortality in this study [4, 5]. The clinical spectrum of COVID-19 is highly heterogeneous, ranging from asymptomatic to acute respiratory distress syndrome and multi-organ dysfunction. SARS-CoV-2 mainly infects the respiratory system, but recent findings have revealed that, besides the respiratory system, the circulatory system, liver, immune system, urogenital system, myocardial, gastrointestinal, renal system, and even the central nervous system could be the target organs for infection [6, 7]. Lymphopenia is one of the common features of COVID-19, which is a decline in the numbers of CD4/CD8 T cells but not B cells. Comprehensive systematic literature searches of online databases have revealed that the significant reduction of T cell count is a hallmark of severe COVID-19. The current results propose that lymphopenia at the initial presentation of COVID-19 is linked poor prognosis in patients [8, 9].

Telomeres are located at the ends of linear chromosomes and are composed of distinct protein elements that bind to telomeric DNA. These nucleoprotein structures protect the chromosomal ends from DNA breaks and preserve the stability of the genome. In most cell types, the continuous shortening of telomeres occurs during cell division due to the incomplete replication of chromosomal ends. Maintenance of telomere length is critical for the efficient proliferation of cells, and therefore telomere length dynamics play a critical role in the biology of diseases [10]. Regardless of chronological aging, several physiological, environmental, and behavioral factors such as smoking, lack of physical activity, obesity, exposure to hazardous air pollutants, stress, childhood adversities, and major depressive disorder can potentially increase the rate of telomere shortening [11, 12].

Leukocyte telomere length (LTL) is a heritable and complex polygenic trait with large inter-individual variation [13]. Genome-wide association studies (GWAS) implicate the involvement of at least eight different gene loci in LTL [14–19]. Based on our present knowledge, short telomeres limit immune cells’ ability to proliferate. There is an interconnecting relationship between shortening of LTL and lymphopoiesis. Telomere attrition to a critical and threshold length can limit the proliferative capacity of immune cells. Shorter LTL is tightly coupled with increased synthesis of proinflammatory cytokines, poorer immune responses to infections, and consequently the development of severe disease [20]. Findings from a cohort study on healthy young and midlife adults demonstrated that shorter CD8CD28− T cell telomere length is associated with increased risk for experimentally induced acute upper respiratory tract infection and clinical illness [21]. Additionally, several studies have highlighted the significant association of shorter telomeres with increased severity of COVID-19 [22–26]. Based on the above, the susceptibility to viral infections with respect to telomere length is not a new idea. Therefore, in this replication study, we attempted to investigate the possible relationship between LTL and the severity of COVID-19 in our population.

## Materials and methods

### Subjects

This study was conducted on 50 intubated adult patients patients, comprising 22 female and 28 male patients with ages ranging from 26 to 91 years old, who were admitted to the ICU with severe COVID-19 infection. Additionally, 50 individuals, comprising 22 female and 28 male patients with ages ranging from 31 to 88 years old, who were hospitalized with moderate COVID-19, were included. The groups were gender matched. Blood samples were collected from May to July 2022, during which the Omicron strain was the dominant variant of the virus in Iran [27].

The key steps involved in identifying and enrolling eligible participants included a positive nasopharyngeal reverse transcription polymerase chain reaction (RT-PCR) test and the presence of symptoms. Participants were 18 years or older. We classified disease severity based on clinical symptoms, breathing problems, and radiological features. Patients with COVID-19 were considered to have severe illness if they had SpO_2_ < 90% on room air at sea level, a respiratory rate > 30 breaths/min, PaO_2_/FiO_2_ < 200 mm Hg, or lung infiltrates > 60. Patients were considered to have moderate illness if they had SpO_2_ < 94% on room air at sea level, a respiratory rate > 25 breaths/min, 200 < PaO_2_/FiO_2_ < 300 mm Hg, or 60 > lung infiltrates > 30. We investigated five comorbid conditions including hypertension (HTN), type 2 diabetes mellitus (T2DM), cardiovascular disease (CVD), chronic obstructive pulmonary disease (COPD) and chronic kidney disease (CKD) in patients with COVID-19.

### DNA isolation and quantitative PCR (q-PCR)

Blood samples were obtained on the first day of admission from all patients. Genomic DNA was extracted from buffy-coat samples using the QIAamp 96 DNA Blood Kit by Qiagen (Hilden, Germany) and stored at − 20 °C. Next, the quantity and purity of the DNA were assayed using a NanoDrop 2000 spectrophotometer (Thermo Scientific, Wilmington, DE, USA), and all DNA samples had OD260/OD280 values of 1.7–2.1. The DNA samples were stored at − 80 °C until further use. The relative measurement of the LTL was carried out by the quantitative polymerase chain reaction (qPCR)-based assay according to a method described by Cawthon [28] with small modifications. A specially designed oligonucleotide primer set hybridized to genomic DNA and amplified telomeric DNA. For accurate and reliable relative telomere length analysis, we normalized the results of the telomere reaction using a specific single-copy gene, 36B4 gene, which encodes an acidic ribosomal phosphoprotein P0 (RPLP0). Briefly, the final reaction mixture contained 5 ng of buffy-coat-derived genomic DNA, 5 µl of SYBR Green master mix (Applied Biosystems), 300 nM of forward and 300 nM of reverse primers, and enough double-distilled water to yield a 10 µl reaction. For each DNA sample, two consecutive reactions were performed with the following primer pairs for the telomeric sequence and housekeeping gene. The list of primers used in this study is summarized in Table 1. The thermal cycle conditions of each qPCR analysis were set at 95 °C for 10 min, followed by 25 cycles of 10 s at 95 °C, 120 s at 56 °C for the amplification of the telomeric sequence, and for 36B4 PCR, followed by 30 cycles of 10 s at 95 °C, 60 s at 58 °C. All assays were carried out in triplicates. Each reaction also contained a 6-point standard curve from 0.625 to 20 ng using pooled buffy-coat-derived genomic DNA. The acceptable standard deviation (s.d.) of the triplicate threshold cycle (Ct) values was set at 0.3. The telomere repeat copy number to single-gene copy number (*T*/*S*) ratio was calculated using the following equation: − ΔCt (ΔCt = CtTel − Ct36B4). The relative average telomere length was determined using the Rotor-Gene quantification software in a Rotor-Gene 3000 real-time System.

**Table 1:** List of primers used in this study

Primer	Sequence(5′→ 3′)
Tel-forward	GGTTTTTGAGGGTGAGGGTGAGGGTGAGGGTGAGGGT
Tel-reverse	TCCCGACTATCCCTATCCCTATCCCTATCCCTATCC-CTA
36B4-forward	CAGCAAGTGGGAAGGTGTAATCC
36B4-reverse	CCCATTCTATCATCAACGGGTACAA

### Statistical analysis

The normal distribution of quantitative data was evaluated using the Kolmogorov–Smirnov test. In the presence of a normal distribution, the correlation between data was assessed using the Pearson test. If the normal distribution was not followed, the Spearman test was used instead. Differences between quantitative data were analyzed using the Mann–Whitney test. The predictive effect of telomere length on the severity of COVID-19 was evaluated using binary logistic regression models with a forward stepwise selection process. All statistical analyses were performed using GraphPad Prism software version 8. A *p*-value under 0.05 was considered significant.

## Results

This article aims to assess the role of telomere length in COVID-19 severity. As expected, we observed a strong inverse correlation between relative LTL and COVID-19 severity. However, our results suggest that there is no significant association between gender, smoking status, and comorbidities in terms of the relative length of telomeres in patients with moderate and severe forms of COVID-19. Our findings indicate a strong negative correlation between telomere length and age of patients with the severe and moderate forms of COVID-19. The present data revealed no significant association between hospitalization duration, occurrence of septic shock, and death with the relative length of telomeres in the subject groups. Finally, related clinical characteristics were compared between two groups of patients with moderate and severe COVID-19 and revealed that there was no significant difference regarding gender, smoking, comorbidities and age (*p*-value = 0.99, 0.79, 0.06 and 0.54, respectively). See supplementary material (Additional file 1: Table S1) for supporting content.

### Telomere length was associated with the severity of COVID-19

**Fig. 1:**
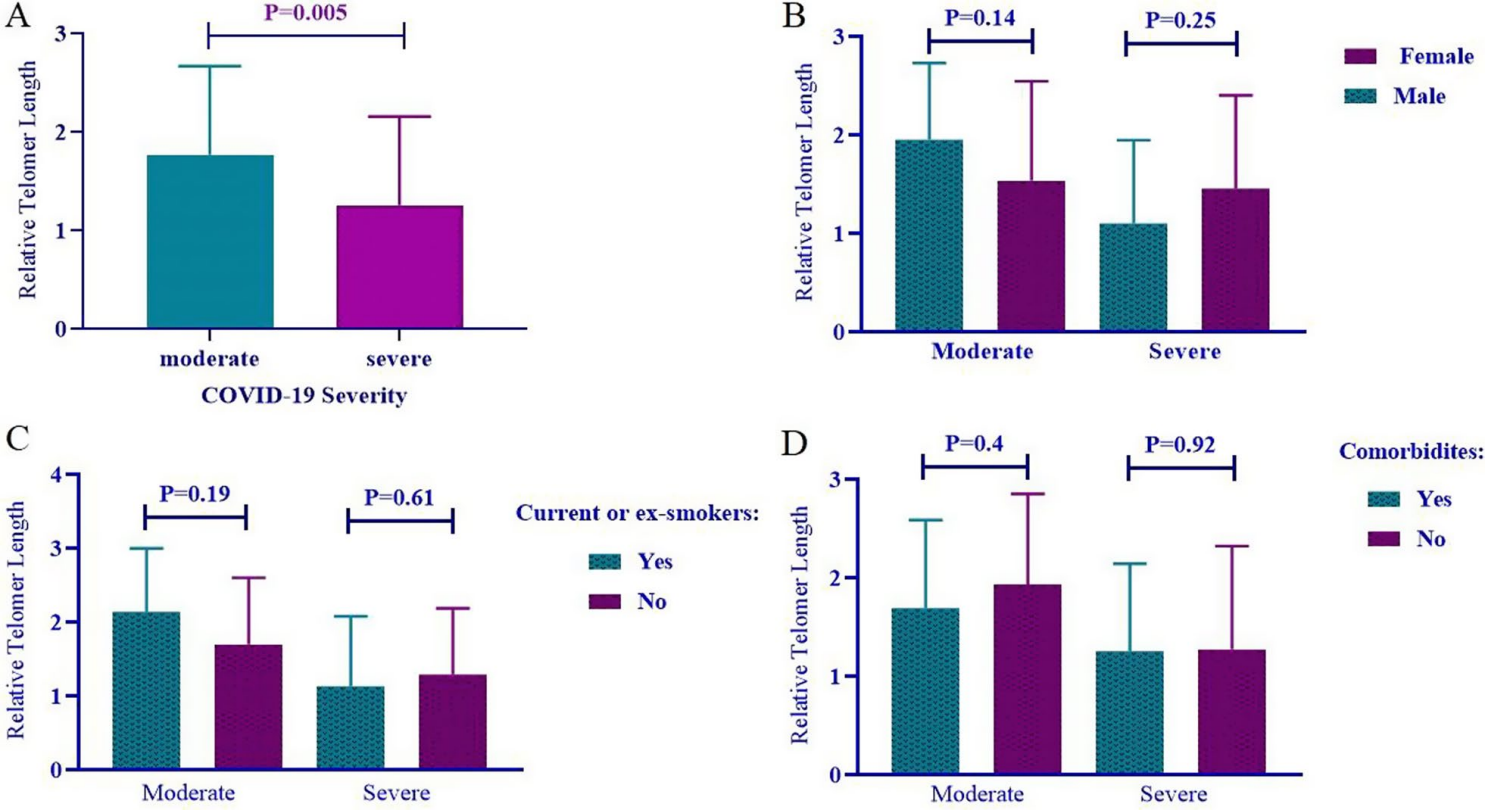
Association of **A** the COVID-19 disease severity, **B** gender of patients, **C** smoking status, and **D** comorbidities with the length of telomeres

To evaluate the association between the severity of COVID-19 disease, gender, smoking status, and comorbidities with the length of telomeres, we used the Student *t*-test. Based on the data (Fig. 1A), the relative length of telomeres in patients with severe form of COVID-19 was significantly shorter than those affected with moderate COVID-19 disease (*p*-value = 0.005).

There was no significant difference between men and women in terms of the relative length of telomeres in patients with moderate and severe forms of COVID-19 disease (Fig. 1B) (*p*-value = 0.141 and 0.259, respectively).

According to our data, no significant association was found between current or ex-smokers and non-smokers regarding the relative length of telomeres in patients with moderate and severe forms of COVID-19 disease (*p*-value = 0.19 and 0.61, respectively; Fig. 1C).

Finally, the investigation revealed no significant association between the length of telomeres and comorbidities in patients with moderate and severe forms of COVID-19 disease (*p*-value = 0.40 and 0.92, respectively; Fig. 1D).

Additionally, logistic regression analysis was performed to evaluate the predicting value of variables regarding the severity of COVID-19 disease. Based on the univariate logistic regression analysis, telomere length and comorbidities were regarded as significant predictors of COVID-19 severity. Multivariate logistic regression analysis showed that telomere length, along with age, had significant predictive values regarding the severity of COVID-19 disease in our study population (Table 2).

**Table 2:** Univariate and multivariate logistic regression analysis to determine the independent predictor of COVID-19 severity

Variable	Univariate	Multivariable	
	*p*-value	OR (95% CI)	*p*-value
Telomere length	0.007	0.22 (0.09–0.51)	0.0001
Age	0.54	0.93 (0.89–0.98)	0.01
Gender(male)	0.99	–	0.29
Current/ex-smoker	0.6	–	0.26
Comorbidity	0.03	–	0.14

### Telomere length was inversely correlated with age in both severity groups

We used the Pearson test for COVID-19 patients with moderate severity due to the normal distribution of age data and the Spearman test for COVID-19 patients with the severe form of the disease to measure the correlation between telomere length, assessed by quantitative PCR, and age of the patients.

**Fig. 2:**
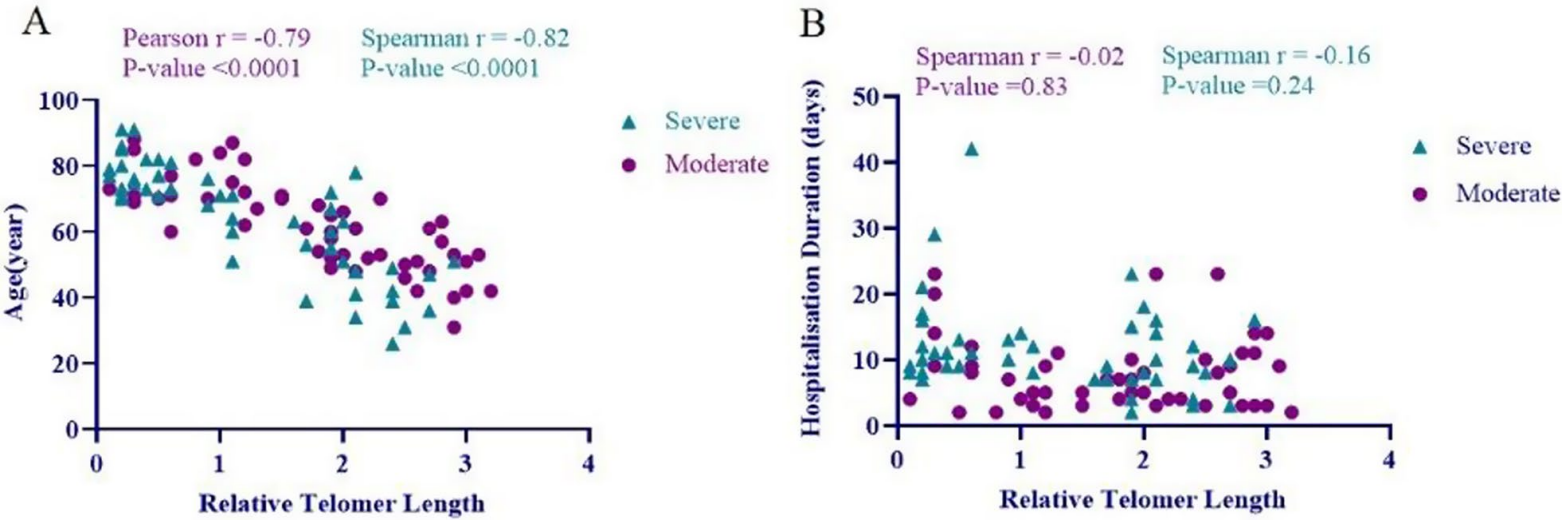
Correlation of **A** age and, **B** hospitalization duration of COVID-19 patients with the relative telomere length with different severity of disease (circle, moderate; triangle, severe)

Our findings revealed a strong negative correlation between telomere length and age of COVID-19 patients with moderate severity (*r* = − 0.79; *p* < 0.0001) (Fig. 2A). The same type of correlation was found between telomere length and age of patients with the severe type of COVID-19 (*r* = − 0.82; *p* < 0.0001). Therefore, the present data showed a significant correlation between the telomeres shortening and the advanced age of the COVID-19 patients, and this correlation was stronger in patients with the severe form of the disease.

Also, evaluating the overall correlation of telomere length with age and length of hospitalization of patients (regardless of disease severity) showed that the age of patients with COVID-19 had a strong and significant inverse correlation with telomere length (*r* = − 0.79, *p*-value = 0.00021), while the duration of hospitalization of COVID-19 patients had a relatively weak inverse but significant correlation with telomere length (*r* = − 0.23, *p*-value = 0.01).

We also evaluated the correlation between telomere length and hospitalization duration in COVID-19 patients with moderate and severe forms of the disease using Spearman analysis. Our results showed that there was no correlation between telomere length and hospitalization duration in moderately affected COVID-19 patients (*r* = − 0.02; *p* = 0.82) and also in patients with the severe form of COVID-19 (*r* = − 0.16; *p* = 0.24) (Fig. 2B).

### Telomere length was not associated with septic shock and death

**Fig. 3:**
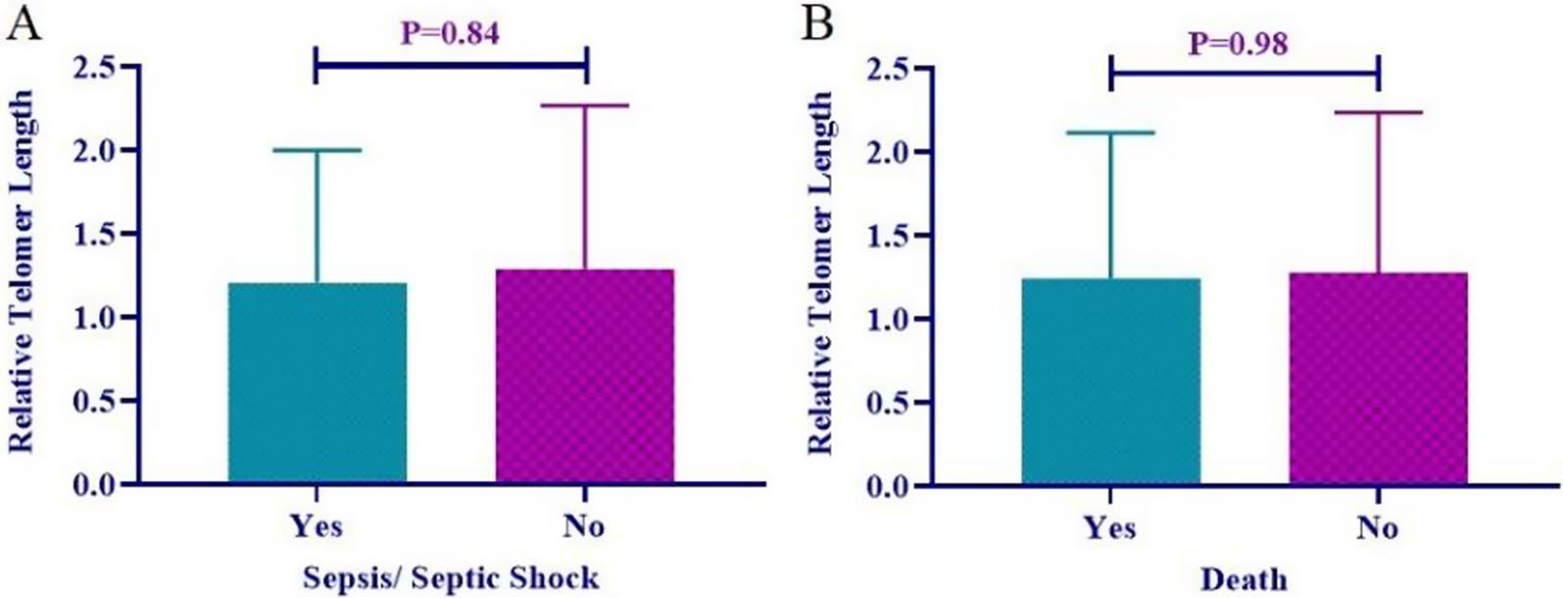
Association of **A** the occurrence of septic shock, **B** death, with the length of telomeres

Finally, we assessed the possible association of telomere length with the occurrence of septic shock and death in patients with the severe form of COVID-19. As shown in Fig. 3, the present data revealed no significant association between the length of telomeres and the occurrence of septic shock (*p* = 0.84) (Fig. 3A), or death (*p* = 0.98) (Fig. 3B), in patients who were affected by the severe form of the disease.

## Discussion

Our approach in this study was to test the association between leukocyte telomere length (LTL) and the severity of COVID-19 in 100 Iranian patients. These findings showed that the telomere length in patients with a severe form of COVID-19 is significantly shorter than those with a moderate form of the disease.

The nine tentative hallmarks of aging include genomic instability, telomere attrition, epigenetic alterations, loss of proteostasis, deregulated nutrient sensing, mitochondrial dysfunction, cellular senescence, stem cell exhaustion, and altered intercellular communication. Telomere attrition or shortening is one of the most well known of these hallmarks. Chronological and biological aging, as well as environmental factors, can affect the rate of telomere attrition [29]. Immunosenescence is a complex phenomenon characterized by dynamic changes occurring in innate and adaptive immune systems, leading to the accumulation of senescent immune cells, subsequently causing the progressive decline in immune system function [30]. Increased levels of circulating pro-inflammatory cytokines [31], reduced percentage of naive lymphocytes [32], and telomere shortening [33] are the main age-associated changes in the immune system and play pivotal roles in reducing immune competency in humans. According to our current knowledge, the cytokine storm is associated with severity and death in COVID-19 patients [34]. Although the promotion of massive lymphopoiesis is crucial for the successful clearance of viral infections, a drastically reduced number of circulating lymphocytes, lymphopenia, has been introduced as a reliable prognostic marker for COVID-19 severity [35]. The survival, proliferation, and maturation of lymphocytes in bone marrow, thymus, and secondary lymphoid organs are tightly coupled with telomere length [36]. Longitudinal follow-up studies on telomere shortening have revealed a heterogeneous pattern in the rate of telomere shortening among individuals [37, 38]. Short telomeres in leucocytes are associated with immune dysfunction and many chronic diseases. Experimental exposure to rhinovirus in healthy young and midlife adults showed a significant association between shorter CD8CD28− cell telomere length and upper respiratory tract infection and clinical illness [21]. Based on a simplified hypothesis, Aviv discussed the possible role of LTL in COVID-19 severity and mortality [39]. Our recent study suggested that telomere attrition and epigenetic alterations are the main predictors of biological aging and cellular senescence that can influence the severity of COVID-19 [40]. A recent study showed a significant association between LTL and lymphopenia among elderly patients with COVID-19 [41]. Two separate studies on LTL in patients demonstrated that the risk of developing severe COVID-19 is higher in individuals with short telomere length in peripheral leukocytes [23, 26]. Additionally, a comparison of LTL in 53 individuals without COVID-19 and patients with different clinical severity showed that patients with the severe form of COVID-19 had the shortest LTL. In comparison with the subjected individuals without COVID-19 and mild groups, patients with moderate COVID-19 had a shorter LTL. No significant differences were reported in LTL between negative and mild groups [42].A cohort study on 6775 participants in the UK Biobank demonstrated the association between shorter LTL and a higher risk of developing severe COVID-19 [25]. In our study, we confirmed a significant inverse correlation between relative LTL and age of COVID-19 patients. Severe patients showed a stronger inverse correlation between relative LTL and age compared to moderate patients. There is also a significant association between relative LTL and COVID-19 severity. According to these findings, individuals with short telomeres have a higher risk of developing severe COVID-19.

Several studies have shown that baseline LTL varies with sex and genetic background. Males have shorter overall LTL and higher attrition rates [43, 44]. However, in our study, there was not enough statistical evidence to show sex-specific associations between telomere length and moderate and severe COVID-19 patients. The differences in methodology (Flow-FISH or qPCR) or statistical analysis (quantile or linear regression) may explain the controversial results of the median LTL between sexes. Therefore, further research needs to be done to better understand this phenomenon [45].

Despite the biological links, smoking habits can affect LTL dynamics among individuals. A systematic review of 84 studies showed that ever smokers had shorter telomeres compared to those who never smoked. Current smokers also had shorter telomeres compared to former smokers [46]. A recent random-effects meta-analysis introduced smoking as a risk factor for the progression of COVID-19; therefore, smoking-control strategies can be effective in blunting the COVID-19 pandemic [47]. However, based on our findings, there was no statistically significant association between smoking and LTL in either moderate or severe COVID-19 patients. Further research on all clinical spectrums of COVID-19, ranging from asymptomatic to mild, moderate, severe, and critical disease, is needed. The results of two different studies suggested that telomere length is associated with a greater risk of developing a critical disease, such as being admitted to ICU or death without ICU. These studies indicated that shorter LTL can influence the severity of COVID-19 [23, 42]. However, in a recent study analyzing telomere length in a cohort of severe hospitalized COVID-19 patients, the role of telomere length was supported as a potential risk factor for hospitalization in COVID-19, but not for in-hospital complications nor persistent post-COVID-19 manifestations [48]. In the present study, we did not detect a significant association between LTL and hospitalization duration or more severe outcomes such as the occurrence of septic shock or death, in hospitalized patients with moderate and severe forms of COVID-19. Therefore, the number of people we included in the study was too small to provide convincing evidence, and further comprehensive research can provide more precise estimates of the effects of LTL on hospitalization duration, the occurrence of septic shock, or death.

A recent bidirectional Mendelian randomization study revealed that there is no significant association between LTL and COVID-19 [49]. The limitations of this study may explain these controversial results. The current research has only focused on hospitalized COVID-19 patients with moderate to severe disease without any specific trends in age, sex, or race/ethnicity. Additionally, the differences in methodology or statistical analysis can affect the results. Therefore, these limitations can serve as an opportunity to identify new gaps, and consequently, new research. Although the power of the main hypothesis of the study was acceptable, the results of the subgroup analysis might be influenced by the relatively low power of the study in subgroups.

## Conclusion

The assessment of leukocyte telomere length (LTL) in moderate and severe COVID-19 patients revealed that telomere length was associated with the severity of COVID-19. However, it was not associated with smoking status, hospitalization duration, or clinical implications such as the occurrence of septic shock or hospital mortality. This study paves the way for further investigations on LTL as a potential prognostic factor for COVID-19. Understanding the causes of inter-individual variations in COVID-19 outcomes can provide new insights into potential targets of telomerase activation and personalized therapeutic interventions.

## Supplementary Information

**Additional file 1**. **Table S1**: Clinical characteristics were compared between two groups of patients with moderate and severe COVID-19, and revealed that there was no significant difference regarding age, gender, comorbidities, and smoking.

## Data Availability

Data and materials are available upon request.
