# Supplementary material for: Association between leukocyte telomere length and COVID-19 severity
Source: Egypt J Med Hum Genet. 2023 May 29;24(1):37. doi: 10.1186/s43042-023-00415-z (PMC10225776; doi:10.1186/s43042-023-00415-z)
Supplement: Supplementary file 1 — Additional file 1. Table S1: Clinical characteristics were compared between two groups of patients with moderate and severe COVID-19, and revealed that there was no significant difference regarding age, gender, comorbidities, and smoking. [file 43042_2023_415_MOESM1_ESM.docx]

**Supplementary**

**Table S1:** Clinical characteristics were compared between two groups of patients with moderate and severe COVID-19, and revealed that there was no significant difference regarding age, gender, comorbidities, and smoking.

| P-value | Severe (N=50) | Moderate (N=50) | Characteristic |
| --- | --- | --- | --- |
| 0.54 | 63.56±16.52 | 61.72±13.3 | **Age (Years) (Mean±SD)** |
| 0.99 | 28/22 | 28/22 | **Sex (Male/Female)** |
| 0.06 | 7/43 | 16/34 | **Comorbidities^1^ (Yes/No)** |
| 0.79 | 10/40 | 8/42 | **Current or ex-smokers (Yes/No)** |

^1^Including hypertension (HTN), type 2 diabetes mellitus (T2DM), cardiovascular disease (CVD), chronic obstructive pulmonary disease (COPD) and chronic kidney disease (CKD).
